# Study on the Influence of Magnesite Tailings on the Expansion and Mechanical Properties of Mortar

**DOI:** 10.3390/ma16227082

**Published:** 2023-11-08

**Authors:** Feifei Jiang, Juan Zhou, Zhongyang Mao, Bi Chen

**Affiliations:** 1 School of Civil Engineering, Nantong Institute of Technology, Nantong 226000, China; 999620140019@just.edu.cn; 2 Suzhou Institute of Technology, Jiangsu University of Science and Technology, Zhangjiagang 215600, China; 3 School of Business, Nantong Institute of Technology, Nantong 226000, China; 4 College of Materials, Science and Engineering, Nanjing Tech University, Nanjing 211800, China; bichen1900@126.com

**Keywords:** magnesite tailings, MgO expansion agent, mortar, compressive strength, flexural strength, porosity

## Abstract

To reduce the mining of high-grade magnesite and solve the environmental pollution caused by magnesite tailings, magnesite tailings were used to produce MgO expansion agent (MEA), and a detailed study of its performance was carried out in this study. Firstly, the effects of different calcination times on the calcination products, the specific surface area, and the activity of MEA were analyzed. Then, the MEA produced by calcinating at 950 °C for 1 h was taken as the research object, and the effects of its content on the expansion performance, compressive strength, and flexural strength of the mortar were studied. The results showed that the decomposition of magnesite tailings after high-temperature calcination produced MEA, and the longer the calcination time, the lower the activity. The calcined tailings could compensate for the shrinkage of the mortar, and the expansion increased with the increase in curing temperature. What is more, when the content was less than 8%, the hydration of MEA filled the pores and improved the compactness, so the strength of the mortar increased with the increase in the expansion agent content. When the dosage was greater than 8%, excessive expansion increased the porosity, causing harmful expansion of the mortar and damaging its integrity, leading to a decrease in strength. Fly ash reduced the expansion of mortar, and after adding 30% fly ash, the expansion decreased by 20.0–36.1%, and the ability to suppress expansion decreased with the increase in curing temperature.

## 1. Introduction

Cement has been widely used as the raw material for concrete. However, when cement comes into contact with water and reacts, its volume gradually decreases. When shrinkage is constrained, shrinkage tensile stress is generated, leading to structural cracking [1,2,3]. This not only reduces the strength of the concrete but also the bearing capacity of the structure and the durability of the concrete. but also makes it easier for harmful ions to enter the interior of the concrete, greatly reducing its service life, resulting in a huge loss of natural resources and economy. The problem of cracking caused by the shrinkage of cement-based materials has been puzzling engineers and researchers. And as the strength of concrete increases, the fineness of cement gradually decreases, the C_3_S content increases, and the water-to-cement ratio gradually decreases. Although these changes improve the strength of concrete, the increased shrinkage of concrete makes it more prone to shrinkage cracks [4,5,6].

Adding an expansion agent to compensate for the shrinkage of cement by utilizing the expansion generated by its hydration is a widely recognized method [7,8,9]. Currently, the expansion agents commonly used in engineering are sulfoaluminate-type, MgO-type, and CaO-type expansion agents. Different from the other two kinds of expansion agents, the MgO expansion agent (MEA) has many advantages, such as a lower water requirement for hydration, a more stable reaction-generated substance, Mg(OH)_2_, an artificially adjustable expansion rate, and so on. So MEA is widely used in modern concrete [10,11,12]. Since the 1970s, a large number of Chinese researchers have begun to study MEA, which is applied to large-scale buildings such as dams and airport pavement. The shrinkage of this mass concrete is well compensated by the use of MEA with different activities during dry shrinkage and temperature shrinkage, which greatly reduces the shrinkage cracks and improves the durability of the mass concrete [13,14].

Nowadays, the MEA sold on the market is basically produced by calcining high-grade magnesite. However, with the continuous exploitation of non-renewable minerals, environmental pollution and resource depletion issues are becoming increasingly serious. Coupled with the government’s restrictions on the mining of non-renewable mineral resources, the grade of magnesite resources is decreasing [15,16,17,18,19]. At the same time, a large amount of low-grade magnesite is discarded every year. These randomly discarded tailings have caused serious resource waste and also occupied a large amount of land. Therefore, how to reduce the mining of high-quality magnesite and solve the environmental pollution caused by tailings has become a very urgent problem.

Some scholars have attempted to use other methods to produce MEA. Temiz [20] prepared an expansion agent containing MgO using calcined dolomite (CaMg(CO_3_)_2_). However, this expansion agent also contained a large amount of CaO, which had a fast hydration rate and generated a large amount of expansion before the concrete hardened. This expansion could not compensate for the shrinkage of hardened concrete. Gao [21] used industrial by-products (magnesite and serpentine) to produce MgO expansion agents. It was found that the products of industrial by-products after calcination could also produce expansion, which could compensate for the shrinkage of dam concrete. However, compared to the concrete used in buildings and bridges, the strength of dam concrete is not high, and it is not clear whether the expansion agent produced by by-products has the same effect on shrinkage compensation as high-strength concrete (greater than 50 MPa). There are still many concerns about whether the expansion agent produced by by-products will reduce the strength of high-strength concrete. The above research confirms the necessity and feasibility of using other methods to produce expansion agents. But there is still a lack of detailed research on the effects of calcination temperature and time on the mineral composition, specific surface area, and activity of MEA, which restricts the development of industrial by-products to produce expansion agents. The above issues are also likely the main reason for the increasingly scarce and expensive prices of magnesite.

This paper used magnesite tailings from Laizhou (China) to produce MEA, studied the influence of different calcination methods on the performance of expansion agents, and analyzed the influence of tailings on the strength and expansion performance of mortar, which provided a new way to solve the environmental pollution of magnesite tailings. And at the same time, the study can effectively reduce the price of MEA and promote the promotion and utilization of expansion agents.

## 2. Materials and Methods

### 2.1. Materials

#### 2.1.1. Magnesite Tailing

**Figure 1:**
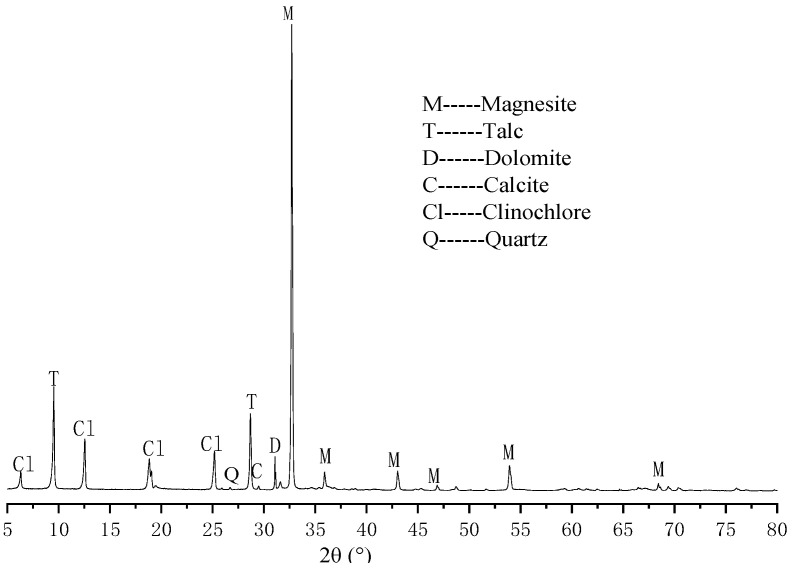
XRD pattern of magnesite tailing.

The magnesite tailings were from Laizhou (Shandong, China), and their chemical composition is shown in Table 1. It can be seen from Table 1 that the content of MgO is lower than 43%, the content of SiO_2_ is higher than 3.5%, and the content of CaO is higher than 1.5%. The main impurities in tailings are chlorite ((Mg,Fe)_4.75_Al_1.25_[Al_1.25_SiO_2.75_O_10_](OH)_8_) and talc (Mg_3_[Si_4_O_10_](OH)_2_), as well as a small amount of dolomite and calcite. According to the classification of magnesite (Table 2), the useful component MgO in tailings is insufficient, while other impurities exceed the standard, making it not one of the four types of ores. It is a low-value tailing with no benefits for mining or processing, so it was often discarded arbitrarily in the past, polluting the environment. The mineral composition of magnesite tailings is shown in Figure 1.

#### 2.1.2. Cement

**Figure 2:**
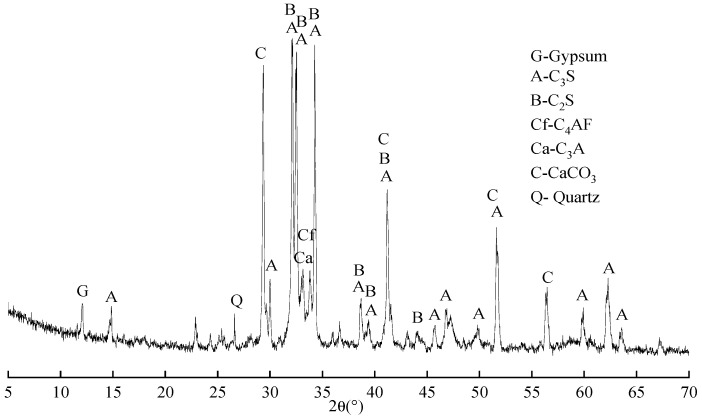
XRD pattern of Portland cement (P·II52.5).

**Figure 3:**
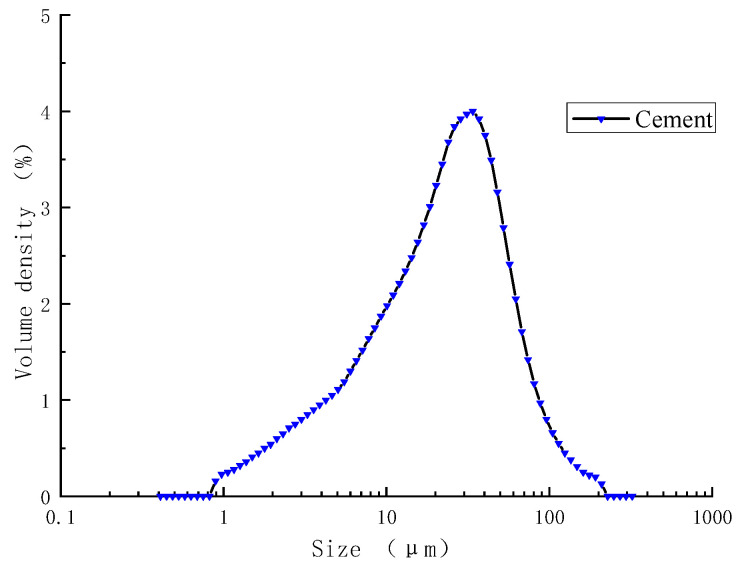
Particle size distribution of cement.

The cement was PII 52.5 Portland cement produced by Jiangnan Xiaoyetian Cement Co., Ltd. (Nanjing, China). Its chemical composition is shown in Table 3, and its mineral composition is shown in Figure 2. It can be seen from Figure 2 that the main mineral composition of cement is C_3_S, C_2_S, C_3_A, and C_4_AF, with a small amount of gypsum and calcite. The particle size distribution of cement is shown in Figure 3.

#### 2.1.3. Fly Ash

**Figure 4:**
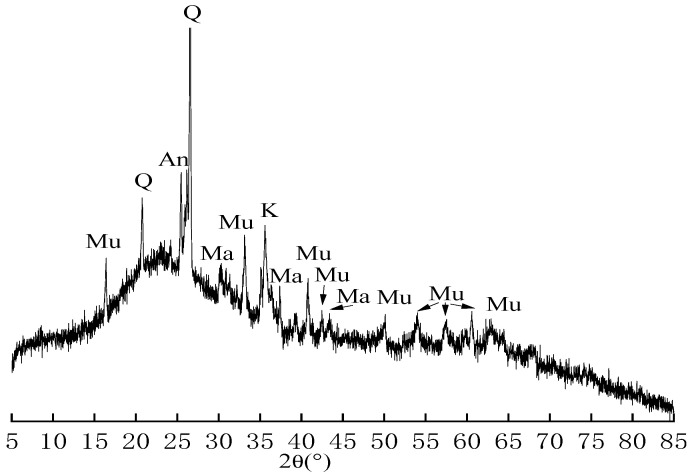
XRD pattern of fly ash: An—Andlusite; Mu—Mullite; Q—Quartz; Ma—Magnetite; K—Kyanite.

**Figure 5:**
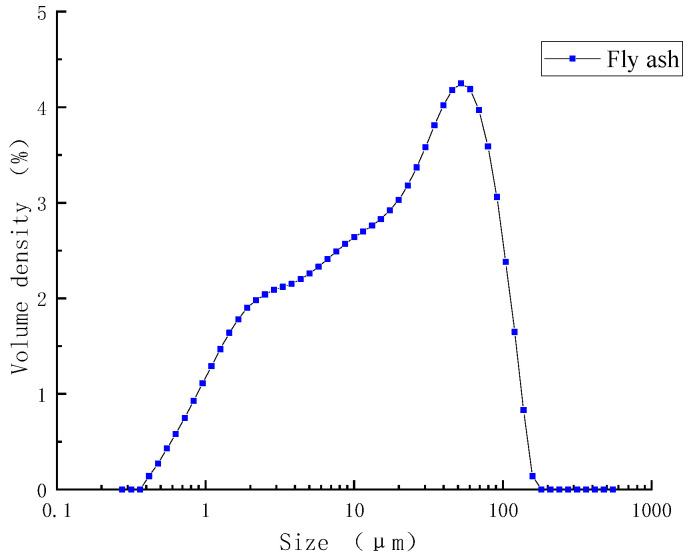
Particle size distribution of fly ash.

Fly ash was class I fly ash from Huaneng Power Plant (Nanjing, China). Chemical composition is shown in Table 4, and mineral composition is shown in Figure 4. The particle size distribution of fly ash is shown in Figure 5.

#### 2.1.4. Sand

**Figure 6:**
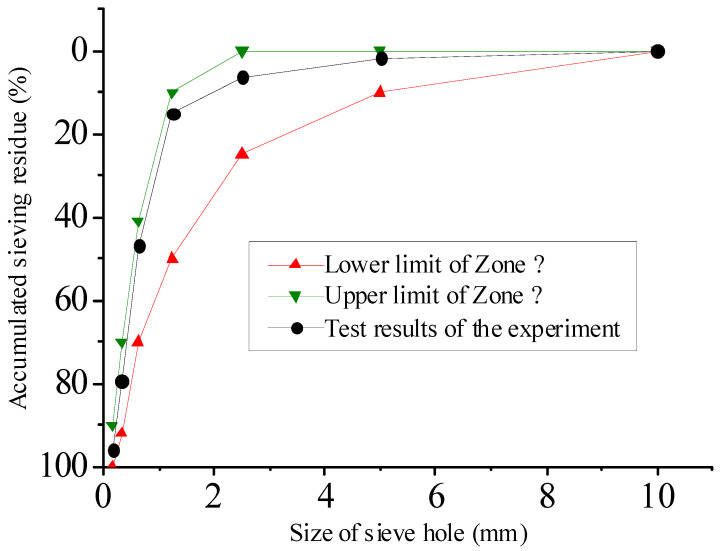
Grading curve of river sand.

The fine aggregate was selected from natural river sand from Nantong (Jiangsu, China). It was tested according to the Chinese standard GB/T 14684-2022 [22], and the test results are shown in Table 5 and Figure 6. The fineness modulus of river sand was 2.1, which belonged to medium sand. The measured cumulative sieve residue of river sand is between the specified upper and lower limits, meeting the grading requirements.

#### 2.1.5. Mix Design

The sand-to-binder ratio (S/B) and water-to-binder ratio (W/B) of mortar were fixed at 3 and 0.5, respectively. Four kinds of mortars with different MEA contents were prepared, and the replacement rates of MEA to cement were 4%, 8%, 12%, and 16%, respectively. In order to study the effect of fly ash on tailing hydration, two different proportions of fly ash (0% and 30%) were used instead of cement.

### 2.2. Experimental Works

#### 2.2.1. Calcination of Tailings

The magnesite tailings were crushed by large, medium, and small jaw crushers in turn. Then they were milled in a mill for 1.0 h and then screened by a 0.016 mm square hole sieve. The method of calcination after briquetting was adopted in the test. After stirring 50 g of sieved tailings with 2.0% water and holding the pressure at 6 MPa for 5 s, a cube of 5 cm × 5 cm × 1 cm was made. Then it was calcined in a box furnace. The calcination temperature was 950 °C, the calcination time was 0.5 h, 1.0 h, 1.5 h, and 2.0 h, respectively, and the heating rate was 6 °C/min. The calcined tailing was cooled rapidly in the air and passed through a square hole sieve of 0.08 mm after being milled for 3 S by a vibration mill.

#### 2.2.2. Performance Test of MEA

(1) Mineral composition

The mineral composition of MEA was analyzed by X-ray diffraction produced in the United States, model ARL XTRA.

(2) Specific surface area of MEA

The specific surface area test of MEA was performed by a specific surface area and micropore analyzer, which is produced in the United States and is model ASAP2020M.

(3) Measurement of f-CaO content

The content of f-CaO in MEA was tested according to the glycerin alcohol substitute method in “Cement Chemical Analysis Method” (GB/T 176-2008 [23]).

(4) Measurement of MgO content

The content of MgO in MEA was tested by X-ray diffraction-phase quantitative analysis (internal standard k-value method). The internal standard material was ZnO, the content was 10%, the scanning range was 35–45°, and the scanning speed was 1 °/min.

#### 2.2.3. Activity of MEA

First, a beaker containing 200 mL of distilled water and 2 drops of phenolphthalein indicator was placed in a constant-temperature water bath at 30 °C. Then, 1.7 g of MEA and some citric acid were placed in a beaker and timed with a stopwatch until the solution turned red. The time recorded by the stopwatch was the active value of MEA. The amount of citric acid is calculated by Formula (1):

*M* = 2.84 × *X* + 2.13 × *Y* (1)

where *M* is the amount of citric acid; *X* is the content of MgO in MEA; and *Y* is the content of f-CaO in MEA.

#### 2.2.4. Mortar Test

(1) Expansion test

The mortar specimen was a cuboid of 40 mm × 40 mm × 160 mm. The effect of MEA on expansion performance was studied by measuring the length variation in mortar specimens. The calcination temperature of MEA was 950 °C, and the calcination time was 1 h. The specimen was demolished after standard curing for 24 h after pouring, and its initial length (*L*_0_) was measured after curing in 20 °C water for 2 h. Then the test specimens were put into water at 20 °C for long-term curing, and the length (*L*_1_) was measured after the corresponding age. Three identical specimens were made for each mix proportion of mortar, and the average expansion of the three specimens was considered the expansion of the mortar. The calculation formula for the expansion rate (*φ*) of the test piece was:

*φ* = (*L*_1_ − *L*_0_)/*L* (2)

where *L* is the initial effective length of the mortar specimen, taken at 150 mm.

(2) Strength test

The strength test was carried out in accordance with the “Test Method for Strength of Cement Mortar” (GBT 17671-1999 [24], China), and the mix proportion was the same as that of the expansion test. Three identical samples were prepared for each dosage in the flexural strength test. Six identical samples were prepared for each dosage in the compressive strength test. The average value of the sample was considered the final strength.

(3) Morphology test

The morphology of mortar containing MEA was examined by SEM (HR-8100, Hitachi, Ltd., Tokyo, Japan).

(4) Porosity and pore size distribution

The porosity and pore size distribution of mortar were measured by a GT-60 mercury intrusion tester produced by Canta Instruments Co., Ltd. (Florida, USA).

## 3. Results

### 3.1. The Effect of Calcination Time on the Calcined Product of Magnesite

**Figure 7:**
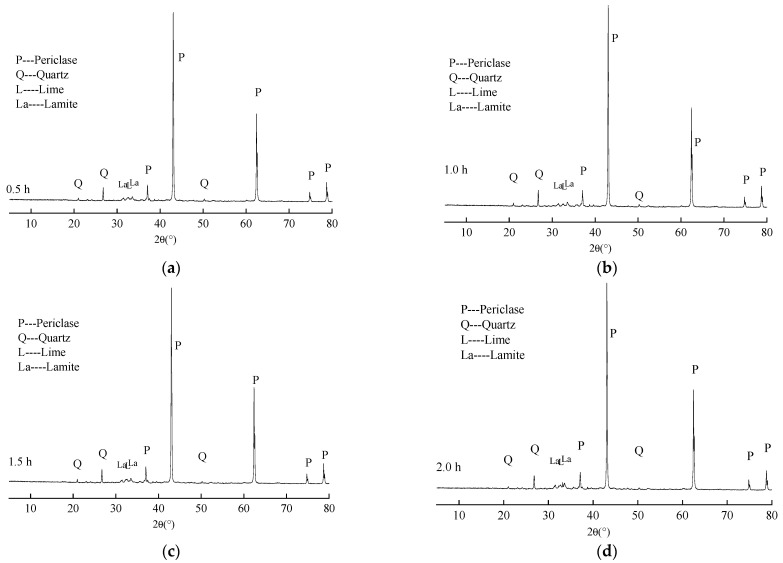
XRD patterns of MEA calcining magnesite tailings at 950 °C for different times: (**a**) 0.5 h, (**b**) 1.0 h, (**c**) 1.5 h, and (**d**) 2.0 h.

Figure 7 shows the mineral composition analysis results of MEA produced by calcining magnesite tailings at 950 °C for 0.5 h, 1.0 h, 1.5 h, and 2.0 h, respectively. From the comparison of minerals before and after calcination in Figure 1 and Figure 7, it can be seen that when the calcination temperature and calcination time were up to 950 °C for 1 h, no magnesite, chlorite, talc, calcite, or dolomite were found in the XRD pattern. This means that there is only a small amount left that cannot be displayed on the XRD pattern. In addition, the XRD pattern shows that the main minerals obtained after 0.5 h of heat preservation were periclase (MgO), as well as a small amount of quartz (SiO_2_), lime (CaO), and lamite (C_2_S). With the prolongation of calcination time, the peaks of MgO, SiO_2_, and C_2_S changed slightly. When the calcination time increased from 0.5 h to 2.0 h, the peak height of MgO increased slightly, the peak height of SiO_2_ and CaO decreased gradually, and the peak height of C_2_S increased obviously.

Table 6 lists the content of MgO in MEA produced by calcining magnesite tailings at 950 °C for 0.5 h, 1.0 h, 1.5 h, and 2.0 h. It can be seen from Table 6 that the content of MgO increased slightly with the prolongation of the calcination time, which indicates that when the calcination time was 0.5 h, there were still a small amount of magnesia minerals that were not completely decomposed. With the prolongation of the calcination time, the decomposition of magnesia minerals was more sufficient, increasing the content of MgO. According to Figure 7 and Table 6, when the calcination time was more than 1.5 h, MgCO_3_, calcite, dolomite, and other minerals in the tailings had almost completely decomposed. Even if the calcination time continued to increase, the content of MgO in MEA would not change greatly.

Table 7 lists the content of f-CaO in MEA produced from calcined magnesite tailings at 950 °C for 0.5 h, 1.0 h, 1.5 h, and 2.0 h. It can be seen from Table 7 that the content of f-CaO decreased with the prolongation of calcination time. This phenomenon, combined with the smaller diffraction peak of SiO_2_ in Figure 7, collectively indicates that as the calcination time increased, f-CaO reacted with SiO_2_ to form C_2_S in a solid state [18].

### 3.2. Effect of Holding Time on Specific Surface Area of MEA

Table 8 shows the specific surface area of MEA produced by calcined magnesite tailings at 950 °C for 0.5 h, 1.0 h, 1.5 h, and 2.0 h. It can be seen from Table 8 that the specific surface area of MEA decreased gradually with the extension of the calcination time. This is because the longer the calcination time, the better the crystallization of MEA and the larger the crystal size, resulting in a smaller specific surface area of MEA.

### 3.3. Effect of Calcination Time on MEA Activity

Table 9 lists the hydration activities of MEA from calcined magnesite tailings with 0.5 h, 1.0 h, 1.5 h, and 2.0 h holding at 950 °C. From Table 9, it can be seen that the activity of MEA decreased with increasing calcination time. This is because the longer the calcination time, the fewer the crystal defects and the smaller the specific surface area of MEA, resulting in a slower reaction rate and thus prolonging the reaction time. Therefore, we can produce different active MEA by changing the calcination time and obtain different expansions to meet the needs of different constructions.

### 3.4. Effect of MEA on Mortar Deformation

**Figure 8:**
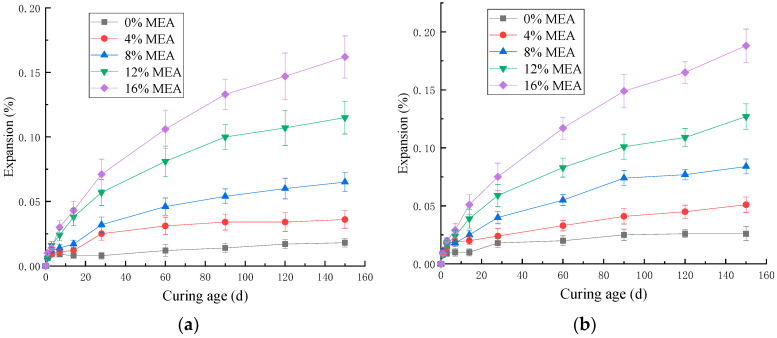
The effect of MEA on expansion of mortars cured in water: (**a**) 20 °C; (**b**) 30 °C.

Figure 8 shows the effect of MEA on the expansion of cement mortar when curing in water at 20 °C and 30 °C. From Figure 8, it can be observed that the expansion rate of MEA mortar was fast in the early stage (within 30 d). Subsequently, as MEA was continuously consumed, its expansion gradually decreased, and there was no phenomenon of expansion regression, indicating that the products after MEA hydration were stable. The increase in curing temperature could promote the hydration of MEA and increase the expansion of mortar. This promoting effect was more obvious in the early stage (within 30 d). For mortar with 8% MEA added, increasing the curing temperature resulted in an increase of 58.3% and 25.0% in expansion after 3 d and 28 d, respectively. This means that during actual construction, we can increase the early curing temperature to improve the ability of MEA to compensate for shrinkage.

**Figure 9:**
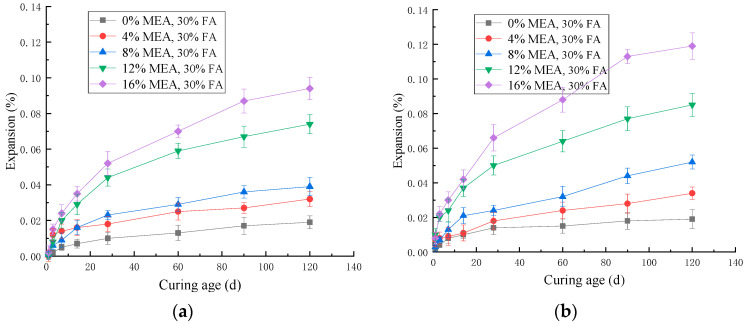
The effect of MEA on the expansion of mortars with 30% fly ash: (**a**) 20 °C; (**b**) 30 °C.

Figure 9 shows the effect of MEA on the expansion of mortar mixed with 30% fly ash. It can be seen from Figure 9 that with the increase in MEA content, the expansion of mortar mixed with 30% fly ash cured in 20 °C or 30 °C water increased gradually. Compared with Figure 8 and Figure 9, the expansion rate of MEA mortar was reduced by adding fly ash. Under the condition of water curing at 20 °C, after adding fly ash, the expansion of mortar mixed with 8%, 12%, and 16% MEA decreased by 35.0%, 30.8%, and 36.1%, respectively, at 120 d. The main reason for this change is that Mg(OH)_2_ reacted with SiO_2_ in fly ash to form C-S-H gel, which consumed the expansion product of MEA hydration, and the generated gel covered the surface of MEA, inhibiting the continued hydration of MEA and thereby reducing the expansion of the mortar [7].

By comparing Figure 8b with Figure 9b, it can be found that after adding fly ash, the expansion of mortar mixed with 8%, 12%, and 16% MEA decreased by 32.5%, 22.0%, and 27.9%, respectively, at 30 °C for 120 d. It can be seen that fly ash inhibited the expansion of MEA, and the degree of inhibition decreased with the increase in curing temperature.

### 3.5. Influence of MEA on Mortar Strength

**Figure 10:**
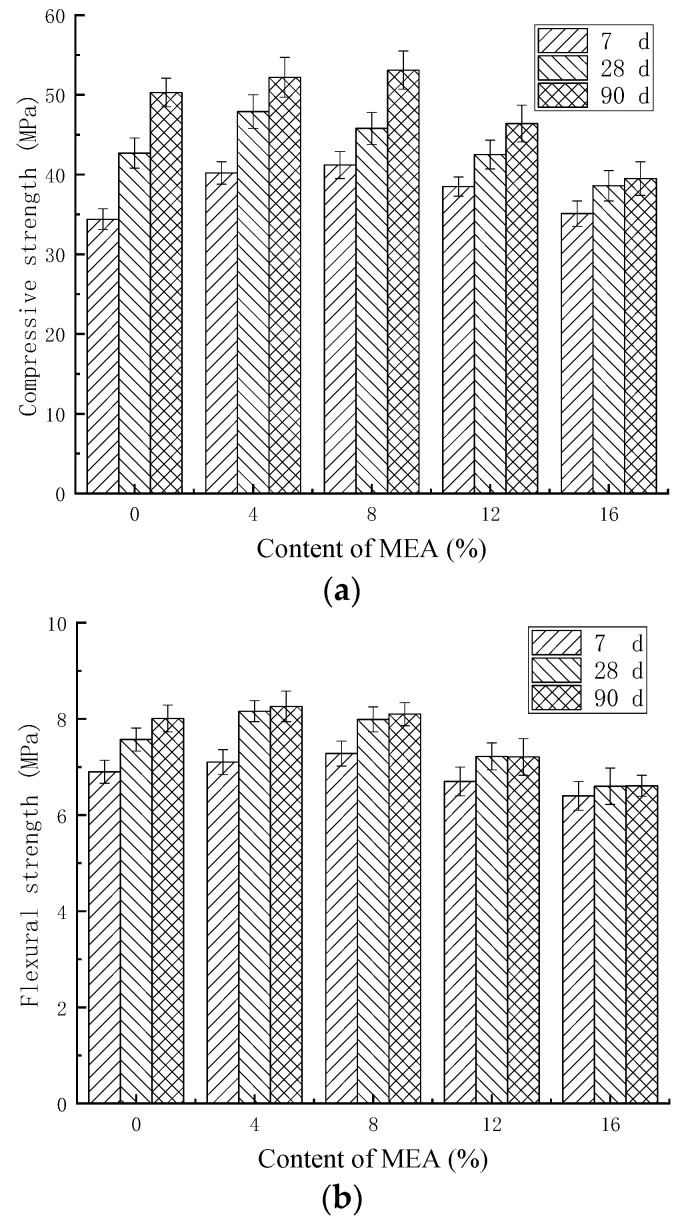
The strength of mortars with MEA: (**a**) compressive strength; (**b**) flexural strength.

Figure 10 shows the influence of MEA on the compressive strength and flexural strength of mortar without fly ash. As can be seen from Figure 10, when the content of MEA was less than 4%, the strength of the mortar increased with the increase in the content of MEA. Compared with ordinary mortar, the compressive strength and flexural strength of 4% MEA mortar increased by 12.18% and 7.94%, respectively, at 28 d. However, when the content of MEA was greater than 8%, the compressive strength and flexural strength of the mortar decreased. And the larger the content of MEA was, the more the strength of the mortar decreased. The compressive strength and flexural strength of mortar with 12% MEA decreased by 7.97% and 9.99%, respectively, at 90 d, and mortar with 16% MEA decreased by 21.64% and 17.48%, respectively. Therefore, from the perspective of ensuring the strength of mortar, the content of MEA should not exceed 8%.

**Figure 11:**
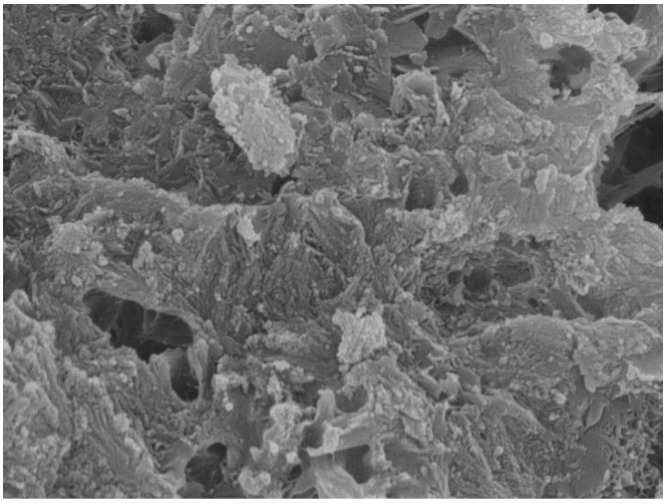
Mortar without MEA at 28 d.

Figure 11 shows the microscopic morphology of mortar without the addition of MEA. From Figure 11, it can be observed that when MEA was not added, due to the shrinkage of the mortar, the structure was loose and the compactness was poor. It can be clearly seen from the picture that there were many small holes. This is also one of the main reasons why the compressive strength and flexural strength of ordinary mortar were not high.

**Figure 12:**
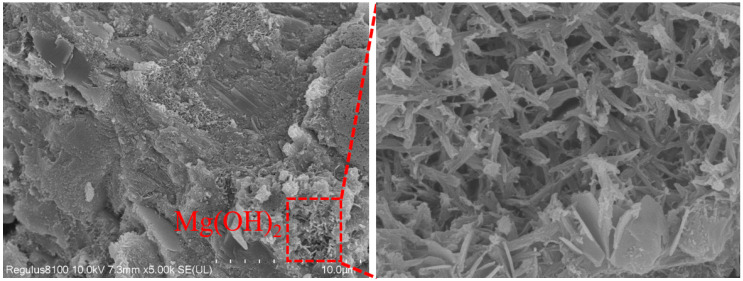
Mortar with 4% MEA at 28 d.

**Figure 13:**
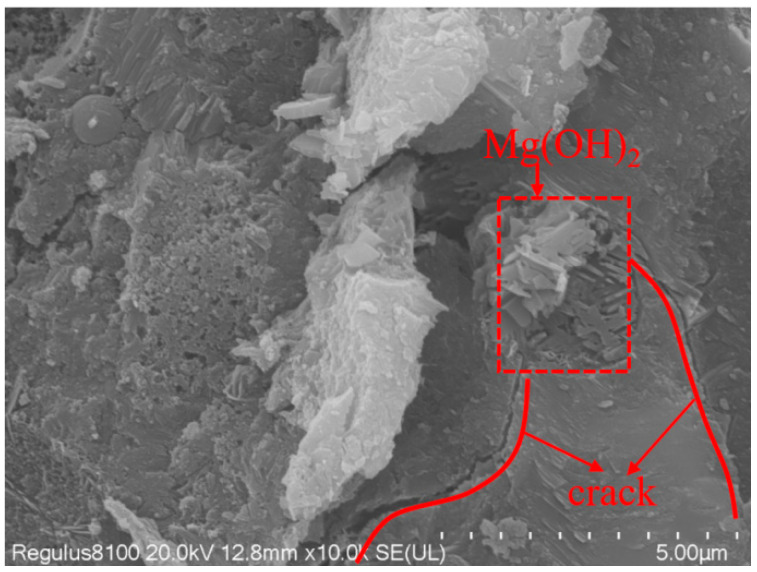
Mortar with 16% MEA at 28 d.

Figure 12 and Figure 13 show the SEM of mortar with 4% MEA and 16% MEA added at 28 d, respectively. From Figure 12, it can be observed that the calcined tailings decomposed to produce MgO. At 28 d, the hydration of MEA generated brucite, resulting in volume expansion. Brucite was in a columnar or sheet-like structure, interwoven together to fill the voids in the concrete, which explains why the strength of the mortar mixed with 4% MEA increased at 28 d. From Figure 13, it can be observed that when the content of MEA increased to 16%, MEA hydration generated a large amount of expansion, which damaged the integrity of the mortar and caused cracks, leading to a decrease in mortar strength.

### 3.6. The Pore Size Distribution and Porosity of MEA for Mortar

**Figure 14:**
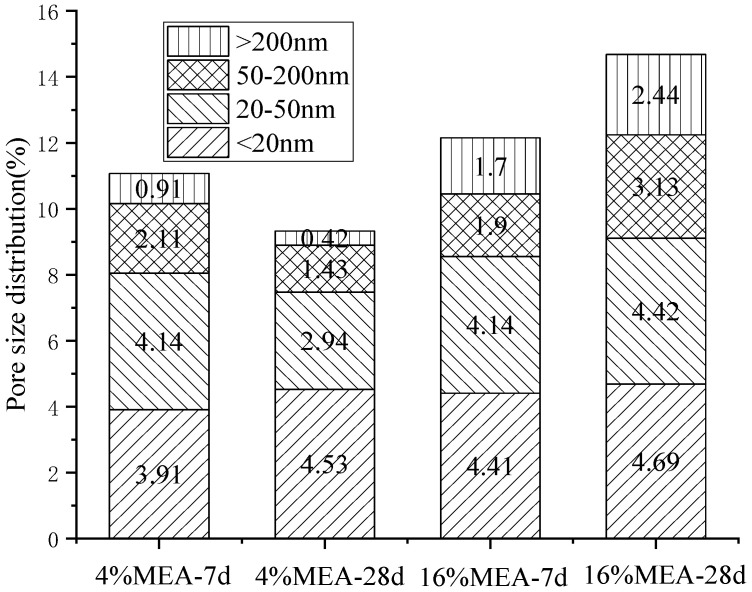
Pore size distribution of mortars containing MEA.

According to ref. [25], the pores in concrete are divided into four categories based on their pore size: harmless pores (less than 20 nm), less harmful pores (20–100 nm), harmful pores (100–200 nm), and more harmful pores (greater than 200 nm). Figure 14 shows the effect of MEA on the pore size distribution of mortar. From Figure 14, it can be seen that when 4% expansion agent was added, as the curing time extended, the expansion agent hydrated and expanded to fill the voids, resulting in a decrease in the total porosity of the mortar, making it denser, especially for larger pores. The porosity at 28 d decreased by 19% compared to 7 d.

However, when the content of MEA increased to 16%, the porosity of the mortar increased by 30.4% at 7 d, and as the curing time increased, the porosity further increased, indicating that excessive expansion caused harmful expansion and damaged the integrity of the mortar. The results of porosity testing are consistent with those of strength testing. When the dosage was less than 8%, the expansion agent compensated for the shrinkage of the mortar, reduced the porosity, and optimized the pore structure. Therefore, the strength increased with the increase in MEA content. The reduction in porosity will also improve the impermeability of concrete, which will improve its durability [26,27,28]. On the other hand, when the dosage reached 16%, excessive MEA would lead to excessive expansion, resulting in more macropores at 28 d compared with pores at 7 d. Therefore, in future engineering applications, it is not only necessary to consider the calcination time of tailings but also to carefully select a reasonable content.

The freezing point of the pore water in the cementitious pore is very low (about −78 °C), which is not easy to freeze and has little influence on frost resistance. Although the freezing point of pore water in macroporous pore is high (about −1 °C), it is not easily saturated and has a buffering effect after freezing, so its impact on frost resistance is also small [29]. Among these three types of pores, the capillary pore has a significant impact on the frost resistance of concrete. It has a freezing point of −12 °C and is easily filled with water. From the pore structure data (Figure 14), after adding 4% MEA, the porosity of the capillary pore decreased at 28 d, indicating that the expansion agent can improve the frost resistance of concrete, which is of great significance for pouring large volumes of concrete in cold cities in Northern China. However, similarly, excessive MEA can also increase the porosity of the pores, so adding expansion agents reasonably is of great significance for improving the durability of concrete.

## 4. Conclusions

Considering the gradual decrease in high-grade magnesite and the environmental pollution caused by magnesite tailings, this paper attempted to use magnesite tailings to produce MEA. In this way, it can not only effectively protect the environment but also reduce the price of MEA and promote the promotion and application of MEA. This paper analyzed the effect of different calcination times on the material properties of MEA and focused on the effect of MEA produced at 950 °C for 1 h on the performance of mortar. The main conclusions are as follows:

(1) Similar to high-grade magnesite, magnesite tailings can also be used to produce MEA with different activities and different expansion properties by changing the calcination time. We can set reasonable production conditions to make use of magnesite tailings, which not only reduces environmental pollution but also compensates for the shrinkage of concrete. The longer the calcination time, the better the crystallization of MEA, the fewer crystal defects, and the smaller the specific surface area of MEA, resulting in a lower activity of MEA.
(2) The expansion of the mortar increased with the increase in MEA content. The early expansion rate was relatively high. With the consumption of MEA, the expansion in the later stage gradually slowed down and tended to stabilize, and there was no expansion regression in the later stage, indicating that the hydration products were stable. Therefore, using calcined magnesite tailings to compensate for concrete shrinkage is effective and safe.
(3) The expansion of MEA increased with the increase in curing temperature, and this increase was more pronounced in the early stage (within 30 d). At 7 d, the expansion of mortar cured at 30 °C increased by 58.3% compared to that cured at 20 °C. Therefore, we can increase the early curing temperature during construction to improve the ability of MEA to compensate for shrinkage.
(4) When the content did not exceed 8%, the strength of the mortar increased with the increase in MEA. In the early stage, the porosity of the mortar was large, and with the increase in curing time, the expansion agent hydration expanded to fill the pores. Compared to 7 d, the porosity of 28 d was reduced by 19%, and the pore structure was optimized, thus improving the strength and durability of the mortar. On the other hand, when the content was greater than 8%, the MEA produced harmful expansion, reducing the strength of the mortar. Especially when the content reached 16%, MEA produced excessive expansion, resulting in the enlargement of the porosity at 28 d, and this harmful expansion led to cracks in the mortar, destroying the integrity of the mortar. Therefore, it is recommended that the content of expansion agents not exceed 8% in engineering construction.
(5) After using MEA and fly ash simultaneously, fly ash inhibited the expansion of MEA. A total of 30% fly ash reduced the expansion of mortar by 20.0–36.1%, and the inhibitory ability decreased with the increase in curing temperature. In order to reveal the underlying reasons for the reduction in expansion caused by fly ash, future research should further analyze the impact of fly ash on the hydration of MEA. In addition, the impact of tailings on the durability of mortar and concrete also needs to be studied. Therefore, the influence of tailings on concrete properties will be discussed comprehensively, which provides a theoretical basis for engineering application.

## Figures and Tables

**Table 1:** Chemical compositions of magnesite tailing.

Material	Chemical Compositions (%)						
	CaO	MgO	Al_2_O_3_	SiO_2_	Fe_2_O_3_	Loss	Total
Magnesite tailing	3.91	41.67	2.45	5.67	0.93	45.13	99.76

**Table 2:** Magnesite classification.

Grade	Chemical Compositions (%)		
	MgO	CaO	SiO_2_
Top Grade	≥47	≤0.6	≤0.6
Grade 1	≥46	≤0.8	≤1.2
Grade 2	≥45	≤1.5	≤1.5
Grade 3	≥43	≤1.5	≤3.5

**Table 3:** Chemical compositions of cement.

Material	Chemical Compositions (%)							
	CaO	MgO	Al_2_O_3_	SiO_2_	Fe_2_O_3_	SO_3_	Loss	Total
Cement	64.73	0.89	4.39	19.41	2.97	2.59	2.40	97.38

**Table 4:** Chemical compositions of fly ash.

Material	Chemical Compositions (%)						
	MgO	CaO	SiO_2_	Al_2_O_3_	Fe_2_O_3_	Loss	Total
Fly ash	0.92	4.40	47.84	34.71	7.31	3.02	98.20

**Table 5:** The properties of river sand.

Apparent Density (g/cm^3^)	Bulk Density (g/cm^3^)	Porosity (%)
2.63	1.51	43.5

**Table 6:** Contents of MgO in MEA at 950 °C for 0.5 h, 1.0 h, 1.5 h and 2.0 h.

Material	Content of f-MgO (%)			
	0.5 h	1.0 h	1.5 h	2.0 h
MEA	73.33	73.48	73.60	73.68

**Table 7:** Contents of f-CaO in MEA at 950 °C for 0.5 h, 1.0 h, 1.5 h, and 2.0 h.

Material	Content of f-CaO (%)			
	0.5 h	1.0 h	1.5 h	2.0 h
MEA	0.94	0.88	0.82	0.56

**Table 8:** Specific surface area of MEA at 950 °C for 0.5 h, 1.0 h, 1.5 h, and 2.0 h.

Material	Specific Surface Area (m^2^·g^−1^)			
	0.5 h	1.0 h	1.5 h	2.0 h
MEA	22.34	20.36	19.45	18.76

**Table 9:** Hydration activity of MEA at 950 °C for 0.5 h, 1.0 h, 1.5 h, and 2.0 h.

Material	Hydration Activity (s)			
	0.5 h	1.0 h	1.5 h	2.0 h
MEA	104	153	187	203

## Data Availability

All data generated or analyzed in this research were included in this published article. Additionally, readers can access all data used to support the conclusions of the current study from the corresponding author upon request.
